# Clinical and radiological outcomes after management of traumatic knee dislocation by open single stage complete reconstruction/repair

**DOI:** 10.1186/1471-2474-11-102

**Published:** 2010-05-27

**Authors:** Michael T Hirschmann, Nadia Zimmermann, Thomas Rychen, Christian Candrian, Damir Hudetz, Lukas G Lorez, Felix Amsler, Werner Müller, Niklaus F Friederich

**Affiliations:** 1 Department of Orthopaedic Surgery and Traumatology, Kantonsspital Bruderholz, Bruderholz, CH-4101, Switzerland; 2 Faculty of Medicine, University of Basel, Basel, CH-4001, Switzerland; 3 Department of Surgery Ospedale Civico, Via Tesserete, Lugano, CH-6903, Switzerland; 4 Department of Traumatology, University Hospital Zagreb, Croatia; 5 Amsler Consulting, Biel-Benken, Switzerland

## Abstract

**Background:**

The purpose of our study was to analyze the clinical and radiological long-term outcomes of surgically treated traumatic knee dislocations and determine prognostic factors for outcome.

**Methods:**

Retrospective consecutive series of patients treated surgically for traumatic knee dislocation with reconstruction/refixation of the anterior (ACL) and posterior cruciate ligaments (PCL) and primary complete repair of collaterals and posteromedial and posteromedial corner structures. 68 patients were evaluated clinically (IKDC score, SF36 health survey, Lysholm score, Knee Society score, Tegner score, visual analogue scale - VAS pain and satisfaction, Cooper test) and radiologically (weight bearing and stress radiographs) with a mean follow up of 12 ± 8 years. Instrumented anterior-posterior translation was measured (Rolimeter, KT-1000). Pearson correlation and stepwise regression analysis was used.

**Results:**

82% of patients (n = 56) returned to their previous work. At final follow-up 6 patients (9%) suffered from pain VAS > 3. The mean side-to-side difference of anterior/posterior translation (KT-1000, 134N) was 1.6 ± 1.6 mm and 2.6 ± 1.4 mm. Valgus and varus stress testing in 30° flexion was <3 mm (normal) in 57 patients (86%). The IKDC score was normal/nearly normal in 38 (58%) patients and the mean Lysholm score 83 ± 17 (intact 98 ± 7). The median Tegner score decreased from 7 preinjury (range 3-10) to 5 at follow-up (range 0-10). The mean Knee Society score was 187 ± 15 (out of maximum 200). In 7 patients (10%) a secondary ligament reconstruction was performed. Three patients (4%) underwent a high tibial osteotomy and four (6%) received a primary unconstrained total knee replacement. According to the Kellgren Lawrence osteoarthritis score only mild degenerative changes were present. The stress radiographs showed stable results for anteroposterior translation. Injury of the lateral collateral ligament, refixation of the ACL/PCL and delayed surgery >40 days were significantly associated with worse outcome (p < 0.05).

**Conclusions:**

Early complete reconstruction can achieve good functional results and patient satisfaction with overall restoration of sports and working capacity. Negative predictive factors for outcome were injury pattern, type of surgical procedure and timing of surgery.

## Background

Traumatic dislocation of the tibiofemoral joint is considered to be rare in Europe and western civilized countries[1,2], but when present it often has dramatic social and economic consequences for the patient[3-9]. Spontaneous reduction makes the true frequency of knee dislocation unclear. Radiological imaging, particularly MRI, regularly underestimates the severity of this injury[10,11]. Dislocation inevitably results in a severe ligamentous injury because of the complex anatomy of the knee joint. However, the exact injury pattern is still a matter of controversy. In fact, traumatic knee dislocation typically leads to injuries of both cruciate ligaments[12], but there have been a few reports of patients with a documented knee dislocation and injury to only one cruciate ligament[13-16]. A tear of both cruciate ligaments with injury to the medial or lateral corner is considered to be a reliable indicator of sustained but spontaneously reduced traumatic knee dislocation[17].

There are a few, predominantly retrospective studies that include a small number of cases, which often address epidemiology, diagnosis and treatment strategy for traumatic knee dislocations in a heterogenous group of patients[8,9,17-34]. Very little knowledge is available on the clinical and radiological long-term outcomes after treatment of these severe ligament injuries. The question of an optimal treatment concept has not been answered adequately. The purpose of our study was to analyze the clinical and radiological outcomes of traumatic knee dislocations treated surgically between 1980 and 2006 in our institution according to a consistent treatment philosophy and to determine factors that predict a better or worse outcome. Our hypothesis was that good results could be achieved in the majority of patients by the implementation of our standardized surgical approach and postoperative protocol.

## Methods

**Figure 1:**
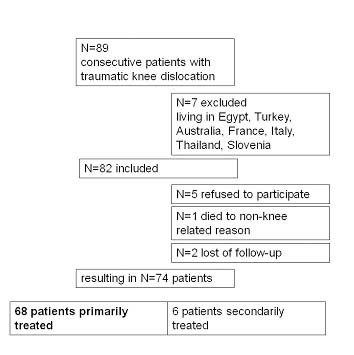
**Patient selection**.

A review of the medical records in the hospital archives was conducted. This yielded 89 consecutive patients with a sustained knee dislocation who were treated surgically between January 1980 and August 2006. Only patients with a radio- or photographically documented knee dislocation or bicruciate ligament injury and associated injury to at least one collateral ligament were included. Exclusion criteria and patient selection are shown in Figure 1. Ultimately, 74 patients were finally included in the present study. Of these 68 patients had received primary surgery and 6 patients secondary surgery (Fig 1.). To homogenize the study population only the results for patients initially treated at our institution were described. Of the patients who received primary surgery 48 were treated within 2 weeks of the trauma and 20 after 2 weeks (within 2-4 weeks n = 7, within 4-8 weeks n = 5, >8 weeks n = 8). The study was approved by the local ethics committee (EK 307/06).

### Surgical technique

The treatment philosophy advocating early open complete ligament reconstruction of the central pivot and peripheral lesions is based on the recommendations and expertise of one of the senior authors. A major goal of the surgical reconstruction was the best possible anatomical restoration of joint biomechanics, which, in our opinion, can only be achieved by a complete restoration of the primary and secondary knee stabilizers[35]. Whenever possible surgery was performed 10-14 days after the injury when the soft tissue swelling had resolved and range of motion had been regained. However, not every patient presented at our clinic at that point in time.

Initially, diagnostic arthroscopy of the knee was undertaken to evaluate the degree of meniscal, cartilaginous and ligamentous injury. A maximum water pressure of 20 mmHg was used and the lower leg was continuously monitored for swelling to preclude compartment syndrome. In cases of meniscal lesion, the damaged tissue was either sutured in outside-in technique with PDS^®^-sutures (Johnson & Johnson, Spreitenbach, Switzerland) or partially removed.

Subsequently, a proximal thigh tourniquet was inflated to 300 mmHg and surgery was continued in open technique. A lateral skin incision and a lateral or medial parapatellar arthrotomy, depending on the pattern of the ligamentous injury, was performed. In cases of a midsubstance tear, the anterior and posterior cruciate ligaments were reconstructed and bone avulsions were managed by screw or suture refixation. Reconstruction of the ACL was performed in anatomical single-bundle technique with ipsilateral patellar tendon autograft. The reconstruction of the PCL was performed in single-bundle tibial onlay technique with ipsilateral quadriceps tendon autograft.

The collateral ligaments and posterolateral/posteromedial corner structures were repaired by insertion of at least three resorbable Z-sutures (Vicryl^® ^2-0, Ethicon, Germany) fixed to a metal anchor (Mitek, DePuy, Spreitenbach, Switzerland). If necessary, popliteal bypass surgery was performed according to the technique described by Müller[35]. All ligaments were tensioned at the end of surgery in the following order: PCL, ACL and finally the collateral ligaments. Detailed descriptions of the reconstruction and refixation techniques have been given previously[35].

Postoperatively the patients were permitted to partial weight bear with an extension splint (3/4 scotch cast splint with 10° extension deficit) from the first week and continued partial weight bearing for a total of six weeks. Limited passive and active, assisted knee flexion was initiated directly after surgery for six weeks. After six weeks the patients progressed to full active knee flexion. The rationale of this protocol was to protect the reconstruction grafts from mechanical stress and allow sufficient motion to prevent arthrofibrosis.

### Follow-up

The median follow-up time was 12 ± 8 years (range 1-27). Demographic data (profession before and after injury, ability to work, need for workers compensation, time to return to work) was noted. Patients were examined by one senior orthopaedic resident who had not been involved in the index surgery.

For clinical outcome assessment we used the International Knee Documentation Committee (IKDC2000) Standard Evaluation Form (demographic, subjective and functional evaluation form)[36-38], the SF36 health survey[39], the Lysholm score[40], the Tegner score[40] and the Knee Society score[41]. The examination included assessment of ACL and PCL laxity with the KT-1000 arthrometer (Medmetric, San Diego, U.S.A.) in 25° flexion with 67N, 89N and 134N and with the Rolimeter (Ormed, Freiburg, Germany) in 25° and 70° flexion. Collateral ligament laxity was tested clinically with varus or valgus stress in extension and 30° flexion. The Cooper asymmetry test (Dial test) was performed in 30° and 90° flexion. In addition, we specified the extent of the patient's subjective perceived impairment on a visual analogue scale (0-10) to evaluate pain (0 = best value and 10 = poorest value) and satisfaction (10 = best value and 0 = poorest value).

### Radiological evaluation

Weight-bearing radiographs of the injured knee (anteroposterior and lateral views), a tangential view of the patella and a Rosenberg view (45° flexion posterior-anterior weight bearing) were obtained. The mechanical alignment of the leg was assessed on full length weight-bearing radiographs. Stress radiographs were obtained with the Telos device (Telos GmbH, Hölstein, Switzerland) in 30° and 90° flexion for both knees to determine anterior and posterior translation.

The medial and lateral joint space height was measured on the Rosenberg view and on anteroposterior weight-bearing radiographs. Any osteoarthritis of the knee joint was graded according to the Kellgren-Lawrence Osteoarthritis score[42]. This score has a five-level scale (grade 0: normal; grade 1: suspected osteoarthritis; grade 2: minimal osteoarthritis; grade 3: moderate osteoarthritis, and grade 4: severe osteoarthritis). All measurements were made precisely using the PACS (Picture Archiving Communication System, Phillips Easy Vision, Netherlands).

### Statistical methods

Data were analyzed using SPSS 13.0 (SPSS, Chicago, U.S.A.). Continuous variables were described using means, standard deviations and ranges. Categorical variables were tabulated as absolute and relative frequencies. Pearson's correlation was used to compute associations between variables. Multivariate influence on several outcome variables was tested by stepwise regression analysis. Variables were entered in four blocks using p < 0.1 as entry criteria and p > 0.2 as removal criteria. Missing values were replaced by the mean. Age at injury, time since injury, gender, physical profession and highest level of education were entered in a first block, Tegner score before injury and smoking in a second block, medial injuries, lateral injuries and peroneal nerve injuries in a third block and type of ACL and PCL surgery in a forth block. Significant variables were marked and adjusted R squares were computed to show the goodness of fit of the multivariate models.

## Results

### Patients and interventions

Patient demographics and injury pattern are shown in Table 1. In 20 patients suture refixation of the anterior cruciate ligament (ACL) and in 48 patients a reconstruction with bone-patellar tendon-bone autograft was performed. In 30 patients (44%) screw refixation of the posterior cruciate ligament (PCL), in 17 patients (25%) suture refixation and in 21 patients (31%) reconstruction with quadriceps tendon autograft was performed. In addition, 6% (n = 4) required partial medial meniscectomy, 4% (n = 3) partial lateral meniscectomy, 22% (n = 15) suture refixation of the medial meniscus, and 31% (n = 21) of the lateral meniscus. 60% (n = 41) had their superficial and/or deep MCL and 31% (n = 21) had their LCL repaired. 14 patients (21%) required a popliteal bypass and in 3 patients (4%) the biceps tendon was reinserted with a metal anchor in the fibula. The peroneal nerve was reconstructed in two patients. No vascular intervention was necessary.

**Table 1:** Patient demographics and injury pattern (n = 68).

Mean age at injury (years)	30 ± 11
**Time since injury (years)**	12 ± 8

**Side of injury (right, left)**	n = 32 (47%), n = 36 (53%)

**Gender (male, female)**	n = 58 (85%), n = 10 (15%)

**Mean height (cm)**	176 ± 8

**Mean weight (kg)**	79 ± 11

**BMI**	25 ± 3

**Insurance status (private, public)**	n = 19 (28%), n = 49 (72%)

**Highest level of education**	

Secondary school graduation	35 (51%)

High school graduation	2 (3%)

Professional training	16 (24%)

University degree	n = 11 (16%)

Post-graduate	n = 1 (1.5%)

Missing	n = 3 (4.5%)

**Profession**	

Physical Non-physical	n = 34 (50%) n = 34 (50%)

**Sports activity prior to trauma**	

*None*	n = 11 (17%)

*only rarely*	n = 33 (50%)

*regularly*	n = 22 (32%)

*no comment*	n = 2 (3%)

**Injury**	

*Sport*	n = 33 (49%)

*motor vehicle accident*	n = 28 (41%)

*work-related injury*	n = 7 (10%)

**Smoking**	n = 33 (49%)

**Injury pattern**	

*ACL*	n = 68 (100%)

*PCL*	n = 68 (100%)

*Superficial MCL*	n = 49 (72%)

*Deep MCL*	n = 44 (65%)

*Medial meniscus*	n = 15 (22%)

*Semimembranosus muscle*	n = 5 (7%)

*LCL*	n = 22 (32%)

*Popliteus tendon*	n = 21 (31%)

*Biceps tendon*	n = 8 (12%)

*Lateral meniscus*	n = 20 (29%)

*Peroneal nerve*	n = 3 (4%)

*Popliteal artery (intimal)*	n = 2 (3%)

### Clinical outcome

82% of patients (n = 56) returned to their previous work. The mean time to return to work was 9 ± 13 months. 10% of patients (n = 7) received full workers compensation. At follow-up, 62 patients (91%) had a VAS pain less than or equal to 3. The results for the visual analogue scale for pain, satisfaction, the total SF36 score, active and passive knee flexion and extension, the Lysholm score and the Tegner score are presented in Table 2.

**Table 2:** Subjective and objective outcome at last follow-up.

Outcome parameter	mean ± SD	median (range)
**VAS pain**	1.4 ± 1.6	1 (0-6)

**VAS satisfaction**	8.8 ± 1.4	9 (4-10)

**SF36 score**	81 ± 15	85 (47-100)

**SF36 physical**	50 ± 7	52 (21-60)

**SF36 mental**	54 ± 8	55 (32-65)

**Active ipsilateral knee extension**	2° ± 3°	0° (0-10°)

**Active contralateral knee extension**	3° ± 4°	0° (0-10°)

**Active ipsilateral knee flexion**	127° ± 9°	130° (100°-140°)

**Active contralateral knee flexion**	135° ± 7°	0° (0°-10°)

**Lysholm score injured side**	83 ± 17	87 (24-100)

**Lysholm score uninjured side**	98 ± 7	100 (55-100)

**Relative Lysholm score**	85% ± 17%	90% (24%-100%)

**Tegner score preinjury**	7.0 ± 1.8	7 (3-10)

**Tegner score at follow-up**	4.9 ± 2.4	5 (0-10)

**Knee Society score**	187 ± 15	191 (118-200)

13 patients (19%) presented with an extension deficit when compared to the uninjured side (5° n = 7, 7° n = 1, 10° n = 5). Clinically, 13 patients (19%) presented with a patella infera compared to the uninjured side. During Lachman's maneuver and anterior drawer testing 64 patients (99%) had a firm endpoint. The pivot shift test was normal in 46 (72%), nearly normal in 15 (23%), and abnormal in 3 patients (5%).

The mean anterior laxity in mm measured with the KT-1000 arthrometer was 1.5 ± 1.0 (67N), 2.7 ± 1.5 (89N) and 4.2 ± 2.5 (134N). The mean posterior laxity in mm measured with the KT-1000 arthrometer was 2.0 ± 1.1 (67N), 3.2 ± 2.1 (89N), and 5.4 ± 2.0 (134N). The mean side-to-side difference for anterior and posterior laxity measured with 134N was 1.6 ± 1.6 mm and 2.6 ± 1.4 mm. The anterior translation measured with the rolimeter in 25° flexion was <3 mm in 46 (72%), 3-5 mm in 17 (26.5%) and 6-10 mm in 1 patient (1.5%). In 70° flexion it was <3 mm in 42 (64%), 3-5 mm in 22 (33%) and 6-10 mm in 2 patients (3%). The posterior drawer test with rolimeter in 70° flexion was <3 mm in 44 patients (63%), 3-5 mm in 22 patients (31%), 6-10 mm in 2 patients (3%). Valgus stress testing in 30° flexion was <3 mm in 57 patients (86%), 3-5 mm in 8 patients (12%), 6-10 mm in one patient (1.5%). Varus stress testing in 30° flexion was <3 mm in 57 patients (86%), 3-5 mm in 8 patients (12%), 6-10 mm in one patient (1.5%). Cooper asymmetry testing at 30° showed <6° (normal) difference in 41, 6°-10° (nearly normal) in 13, 11°-19° (abnormal) in 3, and >19° (severely abnormal) in 7 patients. In 90° flexion the difference was <6° in 49 patients, 6°-10° in 8, 11°-19° in 4 and >19° in 3 patients.

59 patients (89%) did not show any donor site morbidity. In 4 patients (6%) kneeling was tender and in 3 (5%) impossible due to pain. The relative length of a one leg hop in comparison to the uninjured side was >90% in 40 (64%), 76-89% in 12 (19%), 50-75% in 4 (6%) and <50% in 7 patients.

The IKDC subscore for range of motion was A (normal) in 27 (40%), B (nearly normal) in 30 (45%), C (abnormal) in 9 (13%) and D (severely abnormal) in 1 patient (1.5%). The IKDC subscore for ligament laxity was A (normal) in 14 (21%), B (nearly normal) in 30 (46%), C (abnormal) in 13 (20%), and D (severely abnormal) in 9 patients (13%). The total IKDC score was A (normal) in 5 (8%), B (nearly normal) in 33 (50%), C (abnormal) in 18 (27%) and D (severely abnormal) in 10 (15%) patients.

### Complications and secondary surgeries

At final follow-up 6 patients (9%) had a poor result suffering from a pain level VAS > 3. 4 patients were not able to flex their knee more than 110°. Three of them underwent at least one secondary surgery. Overall, in 32 of 68 patients (47%) secondary surgeries including screw removals were undertaken during follow-up. In seven (10%) patients a secondary ligament surgery was performed. Four patients (6%) received a primary unconstrained total knee replacement during the follow-up period, namely, at 2, 2, 17 and 23 years after injury. In three patients (4%) a high tibial osteotomy (in one patient medial and lateral) was performed 0.5, 1, 3 and 5 years after injury. Detailed data on these patients is given in Additional file 1.

### Radiological outcome

On the full length weight-bearing radiographs 37 (54%) patients showed a varus, 21 (31%) a valgus and 10 patients (15%) a neutral mechanical alignment. The mean medial and lateral joint space height in the Rosenberg view was 2.8 ± 2.5 mm and 4.4 ± 2.1 mm. The mean medial and lateral joint space height in the weight bearing anteroposterior view was 2.9 ± 2.4 mm and 4.2 ± 1.5 mm. The Kellgren Lawrence score was 0 (normal) in none, I in 47 patients (suspected osteoarthritis), II in 10 patients (minimal osteoarthritis), III in 10 patients (moderate osteoarthritis), and IV in 1 patient (severe osteoarthritis).

The results of the anterior/posterior stress radiographs in 30° and 90° flexion are shown in Table 3.

**Table 3:** Tibial translation (mm) on stress radiographs in 30° and 90° flexion in comparison of injured and uninjured side.

	30° anterior	30° posterior	90° anterior	90° posterior
	**mean ± sd**	**mean ± sd**	**mean ± sd**	**mean ± sd**

**R/L difference**	2.3 ± 15.2 mm	1.8 ± 4.0 mm	-2.7 ± 5.7 mm	4.5 ± 5.5 mm

### Uni- and multivariate correlations

The results for the univariate correlations are shown in Table 4. Age and time since injury correlate with pre- and postinury Tegner score and the need of secondary surgery. Higher education level correlates with better quality of life, less pain and higher injury correlated scores. Medial side injuries have better outcome than lateral side or peroneal nerve injuries. Reconstruction of ACL correlates with better outcome whether reconstruction of PCL does not correlate with any of the outcomes. Multivariate influence on several of the outcome variables is presented in Table 5. The independent variables explain between 9% (SF-36) and 47% (Tegner score) of the different outcome variables. Patient age at injury does not have any influence on outcome, but time since injury correlates significantly with Tegner score and need for secondary surgery. Education is a very strong independent source for better outcome. Medial side injuries have a positive and peorneal nerve injury a negative influence on outcome also in the multivariate regression model whereas type of surgery does not add significant information.

**Table 4:** Univariate analysis of outcome data.

	SF-36	SF-36 physical	SF-36 mental	VAS pain	VAS satis-faction	Knee Society score	Tegner preinjury	Tegner follow up	Tegner change	Lysholm uninjured side	Lysholm injured side	Lysholm relative	Secon-dary surgery	Need for workers com-pensation	Need to change pro-fession
Age at follow up							-0.47***	-0.36**					0.29*		

Age at injury							-0.41***								

Time since injury							-0.25*	-0.42***	-0.33**	-0.24*			0.56***		

Female sex			0.23^t^				-0.30*								

Physical profession															0.32**

Higher education	0.27*	0.35**		-0.32**	0.35**	0.28*	0.21^t^	0.36**	0.27*	0.28*	0.30*		-0.32**	-0.28*	

Tegner preinjury		0.24^t^					-	0.51***					-0.21^t^		

Smoking	-0.23^t^							-0.31*	-0.35**		-0.34**	-0.31*			

Medial side injuries				-0.36**	0.31**	0.31**						0.23^t^		-0.32**	

Lateral side injuries			-0.21t	0.25*		-0.23t									

Peroneal nerve injury	-0.26*		-0.27*	0.35**	-0.32**	-0.29*							0.23^t^	0.4***	

Time between injury and surgery > 40															0.35**

ACL reconstruction	0.23^t^	0.23^t^		-0.22^t^				0.27*	0.27*		0.24^t^		-0.23^t^	-0.21^t^	0.21^t^

PCL reconstruction															

^t^p < 0.1, *p < 0.05, **p < 0.01, ***p < 0.001

**Table 5:** Stepwise regression analysis on several outcome variables: Adjusted R square of the entire model, and significant influence.

	SF 36 total	VAS pain	VAS satis-faction	Knee Society score	Tegner score^&^	Secondary surgery	Need for workers compensation
Adjusted R Square	0.09	0.25	0.21	0.16	0.47	0.41	0.24

**Block 1: Age and gender**							

Age at injury							

Time since injury					-**	+***	

Gender							

**Block 2: Concomitant aspects**							

Physical profession							

Education	+^t^	-**	+**	+**	+*	-**	-*

Tegner preinjury					+***		

Smoking					-**		
**Block 3: Number of injuries**							

Medial side injuries		-**	+**	+**	+**		-*

Lateral side injuries							

Peroneal nerve injury	-^t^	+^t^				+^t^	+*

**Block 4: Type of surgery**							
ACL reconstruction							

PCL reconstruction							

^&^Time since injury and Tegner preinjury explain R^2 ^= .34; ^t^p < 0.1, *p < 0.05, **p < 0.01, ***p < 0.001

## Discussion

This study, to our knowledge the largest consecutive long-term series on the surgical treatment of traumatic knee dislocations, has produced the following major results.

Firstly, on average 12 years after one-stage open complete reconstruction of traumatic knee dislocations 3/5 of patients showed good to excellent subjective and objective results. On average only 10° loss of flexion was observed. 4/5 of patients returned to their previous work but, on average, the activity level reflected in the Tegner score relevantly decreased from injury to last follow-up. The IKDC score was normal or nearly normal in 3/5 of patients and the Lysholm and Knee Society score showed good to excellent results. The instrumented anterior and posterior laxity measurements with the KT-1000 arthrometer demonstrated good to excellent results at mean 12 years after injury. Less than 5% of patients in the present study had an abnormal (>6 mm) anterior or posterior laxity measured with the rolimeter. These results are consistent with the findings of Tzurbakis et al.[31] who found 1.6 ± 1.9 mm and 2.3 ± 1.7 mm of anterior and posterior translation side-to-side difference. Varus und valgus laxity did not appear to be a major problem. It was normal (<3 mm) in 86% of our patients. This finding is consistent with those obtained by others[19]. Clearly, the measured values of anterior-posterior and varus-valgus laxity reflect a stable knee status in the majority of our patients, which was one major goal of our reconstructive treatment protocol.

Comparison of results with other studies on the treatment of knee dislocations is difficult as study populations often differ with regards to age, injury pattern, demographics, body mass index and treatment protocol. Most authors agree that non-surgical treatment with cast immobilization produces inferior results compared to surgical treatment regimens [5,17,29,43]. A variety of surgical procedures both open[8,21,27,29,31,44] and arthroscopy-assisted[19,21] have been reported.

Using a similar open treatment strategy as ours Tzurbakis et al. reported comparable functional results in terms of instrumented anterior/posterior laxity and Lysholm and Tegner scoring for a series of 25 patients with a follow-up of 2-8 years[31]. Richter et al. reported on a series of patients and compared the outcomes of surgically and non-surgically treated patients. They found inferior results in terms of the IKDC and Lysholm and Tegner scores, which may be explained by the high number of polytraumatized patients in their series[17]. As a referral center for severe knee ligament injuries we often see trauma patients after primary stabilization and care in another hospital, which partly explains the low rate of neurovascular injuries in our series.

With an arthroscopy-assisted combined ACL/PCL reconstruction technique Fanelli et al. reported excellent, mildly superior results with regard to the Lysholm score and Tegner activity score, but the follow-up was rather short[19]. Also Harner et al. and Owens et al. found comparable results for the Lysholm score, loss of flexion, and KT 1000 laxity of the ACL and PCL. However, less patients achieved normal or nearly normal on the total IKDC score[20]. Although in recent years there has been a shift toward arthroscopy-assisted techniques, to our knowledge, all these studies have recorded only equivalent or inferior results in terms of subjective and objective outcomes compared to our long-term series. In addition, several injuries such as posterolateral or posteromedial corner injuries, fractures or avulsed tendons cannot or should not be treated arthroscopically. Therefore, we still propose our treatment protocol (including arthrotomy and open surgery) in acute cases of patients with multiple ligament injuries as a valuable treatment option. In our view, the question of which surgical approach the orthopedic surgeon should choose, i.e. open or arthroscopy-assisted, is only of marginal importance. It is far more the experience and teamwork of the surgeons, physiotherapists and nurses involved in the treatment that makes the difference.

Secondly, we believe that in this study stress radiographs in 30° and 90° flexion obtained with the Telos device have, for the first time, shown convincing long-term laxity results for anterior and posterior translation in surgically treated patients after traumatic knee dislocation. To our knowledge, only Fanelli et al. have to date reported comparable findings with a mean side-to-side difference in posterior translation of 3.2 mm in posterior stress radiographs[19]. Our instrumented radiographic stress testing in both anterior and posterior directions confirmed the clinical impression of a stable ACL and PCL in our long-term series of patients.

Controversy persists as to whether early surgical repair of the ligaments decreases the incidence of posttraumatic osteoarthritis compared with non-surgical treatment. In the present study only a mild degree of osteoarthritis was evident in most of the patients treated according to the Kellgren Lawrence Osteoarthritis score. Only 11 patients (16%) showed a Kellgren Lawrence score of grade III or IV, namely moderate or severe osteoarthritis. This was also reflected by our joint space measurements. This is in accordance with Richter et al.[17] who reported mostly mild and moderate osteoarthritic changes after a similar follow-up time. Interestingly, more degenerative changes were found in the non-surgically treated group of patients, which may be attributed to abnormal joint kinematics due to ligamentous instability. In contrast, Almekinders et al. did not find any difference in radiologically visible degenerative changes of the knee joint between surgically and non-surgically treated patients[45].

Thirdly, we had to accept an unavoidable rate of persistent problems, secondary surgeries and reoperations in this heavily injured patient population. Unfortunately, several authors failed to report their rate of secondary surgeries and/or reoperations[21,22,24]. The biggest problem we encountered after reconstructive surgery was continued pain and loss of motion. A pain level VAS >3 was found in 9% of patients. Decreased ability to flex the knee <110° was present in 4 patients. Seven patients (10%) underwent an arthroscopic arthrolysis during follow-up, which is consistent with the literature[20,27,31].

Instability was not really an issue for most of the patients followed-up in our series, which is reflected by the fact that only 7 patients (10%) underwent a secondary ligament reconstruction during the follow-up period. Three of them were a consequence of insufficient stability after the previous surgery. All but one reoperated patient received ACL suturing and/or PCL refixation. The number of patients requiring high tibial osteotomy or with a primary unconstrained total knee arthroplasty was low considering the long-term follow-up.

Fourthly, according to our results injury pattern, timing of surgery and the chosen surgical treatment might play a crucial role in the long-term prognosis.

In the present study, long-term outcome was significantly influenced by the type of injury.

We found that patients with an injury of the LCL and/or the peroneal nerve had a higher need for workers compensation (p < 0.01). Oswald et al.[25] found similar results reporting that patients with an injury on the lateral side had a less favourable outcome than patients with an injury on the medial side.

The timing of surgery significantly influenced patient's need for workers compensation. Generally it depends on the vascular status of the extremity, soft tissue conditions, concomitant injuries, comorbidities and the injury pattern. There is no doubt that an irreducible knee dislocation, dislocations associated with popliteal artery injury, or open dislocations demand emergency surgical management. In all other cases, there is a general consensus to wait with reconstruction until the inflammatory response has subsided and the full range of motion has been regained. The risk of arthrofibrosis, a major adverse event associated with premature reconstruction, is considered to be less if surgery is postponed until 2-3 weeks after injury[28]. Owens et al. advocated a surgical reconstruction within 2 weeks of the injury and Rihn et al. within 3 weeks of the injury[7,26]. Postponement of surgery beyond 3-4 weeks is not recommended as this may result in excessive scarring of the collateral ligaments[7,28].

The answers to the question of whether a ligament should be reconstructed or not vary greatly in the literature. Most authors advocate a one-stage surgical reconstruction of at least both cruciate ligaments[8,19,20]. Only a few authors reported the results of a multistage procedure. In 2002, Ohkoshi et al. reported good range of motion and antero-posterior laxity (KT-1000 manual maximum) after a two-stage surgical approach (first stage 2 weeks after injury: reconstruction of posterior cruciate ligament, second stage three months later: reconstruction of ligaments that have not healed as a result of non-surgical treatment). However, only eight patients were included in this series[24].

The choice of surgical treatment was significantly associated with better or worse outcome. We found that patients who underwent ACL suturing had a less favourable outcome than patients with reconstruction. Our findings are comparable to those obtained by Mariani et al., who found that patients treated by direct repair had less favorable results in terms of laxity and range of motion than reconstructed patients [22]. Wong et al. found that surgical treatment involving the complete repair of all injured structures was superior in terms of IKDC score and antero-posterior laxity when compared to partial repair (unicruciate ligament reconstruction)[33].

The selection of grafts is still based more on surgeon's preference and availability of grafts than evidence[4]. Patellar tendon, quadriceps tendon and hamstring tendons of the ipsi- or contralateral side have been used as autografts for reconstruction in traumatic knee dislocations. Several authors prefer allografts (Achilles tendon, patellar tendon, tibialis anterior tendon, hamstring tendons) as surgical time and donor site morbidity may be decreased in this complex reconstructive setting[19,23,32]. To our knowledge there is no study demonstrating the superiority of allografts in terms of clinical outcome and ligament laxity in comparison to autografts in multi-ligament injured knees. In addition, allografts are hardly available in Europe. Synthetic grafts have occasionally been used in reconstructive surgery in patients with traumatic knee dislocations[24,30]. A series of 20 patients was evaluated retrospectively by Talbot et al. with a minimum follow-up of one year, yielding inferior results in terms of the Lysholm score, range of motion and ligament laxity[30].

We are aware that this investigation is subject to all the problems inherent in a retrospective study setting, but investigating patients treated for traumatic knee dislocations is hardly feasible in a prospective way. As there is no control group we compared our results to the available literature. However, our study has an extraordinary follow-up rate and is a consecutive series of patients treated at our hospital over a period of 27 years. In the present study performing multiple univariate analysis of about 200 correlations might have led to false positive results in about 10 (p < 0.05) or 20 (p < 0.1) cases and hence the results should be interpreted with all due caution.

## Conclusions

On the basis of our results we advocate early single stage complete reconstruction of both cruciate ligaments and all peripheral structures. Suture refixation of the anterior cruciate ligament should be avoided due to inferior long-term outcomes. Although in recent years there has been a shift toward arthroscopy-assisted techniques, we still propose our treatment protocol (including arthrotomy and open surgery) in acute cases of patients with multiple ligament injuries as a valuable treatment option. In our view, the question of which surgical approach the orthopedic surgeon should choose, i.e. open or arthroscopy-assisted, is only of marginal importance. It is far more the experience and teamwork of the surgeons, physiotherapists and nurses involved in the treatment that makes the difference.

## Competing interests

The authors declare that they have no competing interests.

## Authors' contributions

MH set up the protocol, organized ethics approval, carried out the study and drafted the manuscript. NZ participated in the design of the study, the radiological follow-up and helped with the analysis of radiological data. TR participated in the design of the study and clinical follow-up. CC participated in the design of the study and clinical follow-up and helped with the draft of the manuscript. DH participated in the clinical follow-up and helped with the draft of the manuscript. LGL participated in the design of the study and clinical follow-up and helped with the draft of the manuscript. FA participated in the design of the study and performed the statistical data analysis. WM participated in the design of the study and helped with the draft of the manuscript. NFF participated in the design of the study, interpretation of results and helped with the draft of the manuscript. All authors read and approved the final manuscript.

## Pre-publication history

The pre-publication history for this paper can be accessed here:

http://www.biomedcentral.com/1471-2474/11/102/prepub

## Supplementary Material

Additional file 1 Reoperated patients. Click here for file
