# Supplementary material for: Clinical and radiological outcomes after management of traumatic knee dislocation by open single stage complete reconstruction/repair
Source: BMC Musculoskelet Disord. 2010 May 27;11:102. doi: 10.1186/1471-2474-11-102 (PMC2894008; doi:10.1186/1471-2474-11-102)
Supplement: Additional file 1 — Reoperated patients. [file 1471-2474-11-102-S1.DOC]

## Additional file 1 - Reoperated patients

Legends: ACL=anterior cruciate ligament, PCL=posterior cruciate ligament, sMCL=superficial medial collateral ligament, dMCL=deep medial collateral ligament, ML=lateral meniscus, MM=medial meniscus, Semi=semimembranosus muscle, Gastro=gastrocnemius muscle, Popl=popliteus tendon, PatLig=patellar ligament, Peron=peroneal nerve, Biceps=biceps tendon, Femur=femur fracture, Recon=reconstruction, Refix=refixation/suture, Sut=suture, ORIF=open reduction internal fixation, PartMen=partial meniscectomy, PopByp=popliteus bypass, HTO=high tibial osteotomy, TKR=total knee replacement

| **Pat.**  **no.** | **Age injury (yrs)** | **KD** | **FU**  **(yrs)** | **Injury** | **Initial surgery** | **Reoperation (time from 1^st^ surgery)** | **Tegner preinjuryand FU** | **VAS pain FU** |
| --- | --- | --- | --- | --- | --- | --- | --- | --- |
| **Arthrolysis, debridement, implant removal** | | | | | | | | |
| 1 | 28 | 3m | 20 | ACL  PCL  sMCL  dMCL  MM  ML | Refix  Refix  Sut  Sut  Sut  Sut | Arthroscopic debridement, lysis of patellar ligament, tibial screw removal (2 years) | 7/2 | 0 |
| 2 | 43 | 3m | 9 | ACL PCL sMCL  dMCL | Recon Refix  Sut  Sut | Arthroscopic arthrolysis and tibial screw removal (1 year) | 6/6 | 0 |
| 3 | 21 | 3m | 4 | ACL PCL sMCL  dMCL | Recon Refix  Sut  Sut | Arthroscopic arthrolysis and debridement (1 year) | 7/7 | 1 |
| 4 | 21 | 3m | 25 | ACL PCL sMCL  dMCL | Refix  Refix  Sut  Sut | Arthroscopic debridment (9 and 11 years) | 6/6 | 3 |
| 5 | 29 | 4 | 19 | ACL PCL  LCL Popl ML Biceps Gastro | Recon  Refix  Refix , PopByp  Sut  Refix  Sut | Arthroscopic arthrolysis (1 year) | 4/4 | 3 |
| 6 | 24 | 4 | 21 | ACL PCL  sMCL  dMCL  ML Popl | Refix  Recon  Sut  Sut  Refix  PoplByp | Arthroscopic debridement (14 years) | 8/2 | 0 |
| 7 | 36 | 3m | 21 | ACL PCL sMCL  dMCL  Semi | Refix  Recon  Sut  Sut  Sut | Arthroscopic arthrolysis and debridement (1 and 11 years) | 6/6 | 0 |
| 8 | 31 | 3m | 34 | ACL PCL sMCL  dMCL | Recon  Recon  Sut  Sut | Arthroscopic arthrolysis and debridement (6 and 10 years) | 4/2 | 1 |
| 9 | 26 | 3l | 11 | ACL PCL  LCL Biceps Popl Peron | Refix  Refix  Refix  Refix  PopByp  neurolysis | Arthroscopic arthrolysis (0.5 year) | 9/3 | 5 |
| **Meniscus reoperations** | | | | | | | | |
| 10 | 17 | 3l | 4 | ACL PCL LCL Popl ML | Recon  Recon  Refix  PopByp  Refix | Arthroscopic partial medial meniscectomy (1 year) | 9/7 | 2 |
| 11 | 62 | 3l | 18 | ACL  PCL  LCL  ML | Refix  Refix Refix  Sut | Re-suture lateral meniscus, 3x partial medial meniscectomy (2,3,5 and 13 years) | 4/2 | 5 |
| **Ligament reoperations** | | | | | | | | |
| 12 | 22 | 3m | 19 | ACL  PC  sMCL  dMCL | Refix  Refix  Sut  Sut | Reconstruction ACL with quadriceps tendon autograft (8 years) | 7/3 | 1 |
| 13 | 27 | 3m | 13 | ACL PCL sMCL  dMCL | Recon  Sut  Sut  Sut | Arthroscopic assisted re-refixation of PCL with patellar tendon autograft (3 years), 6x debridement and antibiotics due to infection | 9/4 | 3 |
| 14 | 35 | 3m | 27 | ACL PCL sMCL  dMCL  Popl | Recon  Refix  Sut  Sut  PoplByp | Reconstruction of PCL with patellar tendon autograft (2 years) | 3/7 | 0 |
| 15 | 25 | 3l | 3 | ACL  PCL  LCL Popl  Peron | Recon  Refix  Refix  PopByp  neurolysis | Revision peroneal nerve, reconstruction PCL with quadriceps autograft (1 year) | 5/5 | 5 |
| 16 | 17 | 3m | 22 | ACL PCL sMCL  dMCL  MM  Popl | Refix  Refix  Sut  Sut  PartMen | ACL and PCL reconstruction, popliteus bypass (2 years) | 3/2 | 2 |
| 17 | 40 | 3m | 24 | ACL  PCL  sMCL  dMCL  ML  Semi  GastroPopl | Recon  Recon  Sut  Sut  Sut  Sut  Sut | Re-reconstruction of PCL, popliteus bypass, augmentation of ACL (3 years) | 7/2 | 4 |
| 18 | 21 | 3m | 21 | ACL PCL sMCL  dMCL | Refix  Refix  Sut  Sut | Reconstruction of ACL with patellar tendon autograft, augmentation of PCL, popliteus bypass (2 years) and arthroscopic debridement (20 years) | 7/4 | 1 |
| **HTO** | | | | | | | | |
| 19 | 30 | 3m | 7 | ACL  PCL  sMCL  dMCL | Recon Recon  Sut  Sut | HTO medial open-wedge (3 years) | 10/7 | 1 |
| 20 | 25 | 3m | 9 | ACL PCL  sMCL  dMCL  MM | Refix  Refix  Sut  Sut  Sut | HTO medial open-wedge (1 year)  HTO lateral closing-wedge  (5 years) | 9/5 | 0 |
| 21 | 38 | 3l | 11 | ACL PCL LCL ML | Refix  Recon  Sut  Sut | Reconstruction ACL with patellar tendon, HTO with Heidelberg external fixator (0.5 years), debridement due to infection (1 year), arthroscopic debridement (2 years) | 3/7 | 5 |
| **TKR** | | | | | | | | |
| 22 | 42 | 3m | 27 | ACL  PCL  sMCL  dMCL  MM  ML | Recon  Refix  Sut  Sut  Sut  Sut | TKR (23 years) | 3/4 | 0 |
| 23 | 36 | 2 | 9 | ACL PCL Femur ML | Refix  Refix  ORIF  Sut | TKR (2 years) | 3/0 | 3 |
| 24 | 48 | 3m | 28 | ACL  PCL  sMCL  dMCL  Biceps  MM | Recon  Recon  Sut  Sut  Refix  PartMen | TKR (17 years) | 6/3 | 2 |
| 25 | 34 | 3l | 16 | ACL  PCL  LCL  Popl ML  MM PatLigPeron | Recon  Refix  Sut  PopByp  Sut  Sut  Sut  neurolysis | TKR (2 years) | 7/3 | 0 |
